# Opinions of visually impaired adults on the care provided at community pharmacies: a qualitative interview study

**DOI:** 10.1007/s11096-025-01888-1

**Published:** 2025-03-13

**Authors:** Ellen Roche Ryan, Harriet Bennett-Lenane

**Affiliations:** School of Pharmacy, University College Cork, Cavanagh Pharmacy Building, College Road, Cork, Ireland

**Keywords:** Care, Community Pharmacy Service, Patient, Persons, Qualitative Research, Visually Impaired

## Abstract

**Background:**

Adults who are Visually Impaired (VI) often experience challenges in taking medications and interacting with healthcare settings.

**Aim:**

The aims of this study were to (1) explore the opinions of VI adults regarding care provided at community pharmacies in Ireland and (2) identify patient recommendations for improved care.

**Method:**

Semi-structured interviews were conducted using a topic guide via telephone or videoconference with VI adults who visit community pharmacies in Ireland. Interviewees were recruited on a voluntary basis using a mix of purposive, convenience and snowball sampling. Interviews were recorded, transcribed and carried out until theoretical data saturation. The Braun and Clarke approach was used for thematic data analysis using NVivo software.

**Results:**

Four major themes emerged from eighteen interviews. These were staff awareness, medicines information accessibility, physical inaccessibility and positive supports. Interviewees identified how open communication and close professional relationships between staff and VI adults could be influential in overcoming a perceived lack of independence and privacy currently experienced. Need for staff awareness training, improved provision of medicines information, improved physical accessibility of pharmacy layouts and use of mobile application as assistive technologies were also recommended.

**Conclusion:**

This study provides the first qualitative exploration of care provided to VI adults by community pharmacies in Ireland. Based on personal experiences, interviewees recommended mostly minor adjustments to pharmacy practice to increase accessibility and help provide improved care for this cohort when visiting a pharmacy. This work represents a springboard for future research involving bespoke interventions and tailored guidance for pharmacy teams.

**Supplementary Information:**

The online version contains supplementary material available at 10.1007/s11096-025-01888-1.

## Impact statements

- This study identified that deficiencies in communication and failure to identify those who are visually impaired are leading to negative experiences for these patients in community pharmacies.
- Visually impaired patients recommend staff be provided with awareness training and make use of available technology to meet the needs of caring for this cohort in the community pharmacy setting.

## Introduction

Latest estimates approximate the worldwide incidence of moderate to severe visual impairment at 295 million [1]. Like other sensory impairments, those who are Visually Impaired (VI) can live with this disability from birth or it can be acquired. Latest estimates suggest around 90% of cases are acquired [2]. Certainly, this significant proportion of acquired visual impairment, coupled with rapidly aging populations worldwide, suggests that caring for and communicating with VI patients will become increasingly common into the future. VI adults typically experience disparities in health-related quality of life and functioning versus sighted individuals [3]. Despite prevalence of international legislation advocating for the rights of persons with disabilities in provision of goods and services, health inequalities remain [4]. Studies have demonstrated that those with VI are more likely to experience increased difficulty in reporting poor health [3, 5], as well as inadequate access to quality healthcare and preventive services [6, 7]. Ultimately leading to a higher incidence of negative health outcomes. The World Health Organisation has reported that such poorer health outcomes for people with disabilities are due in part to unfair conditions they face in the health system [8].

Regarding general health advice and medicines information, community pharmacists are often the health system point of entry for most patients [9]. Due in part to extended opening hours, proximity, affordability and lack of appointment necessity. Internationally, community pharmacies play an important role in health promotion and medication safety. VI adults are posed with various challenges in taking their medications and interacting with healthcare settings. Previous findings indicate that older VI patients require significantly more support in medication self-management [10]. They are more than twice as likely to require help managing their medicines and four times as likely to receive support from a community pharmacist versus sighted counterparts [11]. Accordingly, it is clear the pharmacist plays an important role in the care of VI patients.

However, despite the significant role of community pharmacies, gaps are evident in the delivery of pharmaceutical care to VI patients, with an apparent lack of routine services and provisions available. Previous international literature concerning both patient and pharmacist views has highlighted multiple barriers to care, a perceived lack of preparedness generally speaking by pharmacy staff to provide care and a mixed or at times, substandard perception of care provided to them by VI patients [2, 12–17]. Literature from western Europe has, up to now, primarily focused on community pharmacy experiences of older adults with sensory impairments in Scotland, while to date nothing in known about the experiences of Irish VI adults of all ages.

According to the 2022 census approximately 297,000 people in Ireland experience blindness and in a typical week over 2.1 million people or approximately half of the adult population visit a community pharmacy [18]. VI adults can visit a community pharmacy setting for lots of different purposes, not just to collect prescription medications. In Ireland, the role of the community pharmacist is experiencing a significant expansion into the future, with the expected introduction of increased responsibilities and improved clinical services [19]. Therefore, given this evolving pharmacy landscape this study presents a timely opportunity to understand the current experiences of adult patients with VI when visiting a pharmacy in Ireland, of which little is currently known.

### Aim

Using semi-structured interviews with VI adults, this study primarily aimed to explore the opinions of VI adults regarding care provided at community pharmacies in Ireland and to identify patient recommendations for improved care.

### Ethics approval

Ethics approval for this study was granted by the School of Pharmacy Social Research Ethics Committee (SREC) in University College Cork (Reference number 2023–014). All participants provided informed consent.

## Method

### Study design and recruitment

This study is reported in line with the Standards for Reporting Qualitative Research (SRQR) checklist [20]. The study adopted qualitative methodology using semi-structured interviews. Little is known about current experiences of VI adults in Irish community pharmacies and the purpose of the study was to explore personal experiences and recommendations of patients. Therefore, semi-structured interviews were adopted as the preferred method of data collection to facilitate comprehensive investigations into individual experiences.

The study focused on adults aged 18 years or older in Ireland, with a visual impairment, i.e., reduction in visual acuity and/or visual field, as classified by the Visual Classification System. This was adopted and provided by Vision Ireland. It is based on the International Blind Sports Federation (IBSA) Classification for Athletes with Visual Impairment [21]. Categories range from B1 to B5, where B1 refers to the greatest extent of VI or “no vision”, and B5 refers to “useful vision”. B1–B3 may be referred to as “legally blind” or “registered blind”. Further information can be found in Supplementary File 1. Interviewees were required to have visited a community pharmacy in Ireland in the six months previous. They were not required to be a recipient of regular prescription medications.

Interviewees were recruited through a mix of purposive, convenience and snowball sampling. Purposive sampling was used to ensure a range of ages and extent of visual impairment of interviewees. On behalf of the research team, an email was disseminated by representatives from Vision Ireland, Ireland’s national sight loss agency, which notified potential participants of the study and contact information. Similar information was included in the Vision Ireland monthly newsletter. Interviewees next contacted the research team to express an interest in participating and a suitable time was arranged to conduct the interview. An information sheet and consent form were provided to interviewees via email in advance of the interviews. This was compliant with accessibility requirements for VI adults and was available in alternative formats (e.g. braille) if requested by participants. All participants provided written informed consent.

### Data collection

The interviews took place between October and December 2023. A topic guide (Supplementary File 2) composed of five main topics was constructed based on a review of existing international literature [12, 15–17]. Topics covered included: overall experience, challenges experienced, staff, accessible materials, and suggestions for improved service (if applicable). The interview guide included main, and subsequent follow-up open-ended questions and prompts to facilitate development of ideas. For validation purposes, representatives from Vision Ireland reviewed the topic guide to ensure appropriateness and accessibility of all questions. Minor alterations were suggested which were implemented. Vision Ireland provided guidance to the team throughout the project on research methodology and procedures (including the ethics application, interview schedules and survey development). In addition, interviewees 1 and 2 were asked to suggest any changes to the topic guide at the end of their interviews. On both occasions no changes were suggested. Data from these interviews were included in the final analysis.

All semi-structured interviews were conducted by ERR in October to December 2023, a pharmacy student with previous experience working in a community pharmacy. Demographic information was obtained prior to the start of the interview. Interviews were conducted either via videoconference (using Microsoft Teams) or via telephone based on interviewee preference. The interviews were audio-recorded (with permission), transcribed verbatim and deidentified.

### Data analysis

The Braun and Clarke approach to inductive thematic analysis was used for data analysis [22]. All transcripts were entered into NVivo Version 12. Data analysis was performed in tandem with data collection. In the initial phase, transcripts were read repeatedly to allow for data familiarisation. Open coding was utilised in generating unranked codes. Initially, five transcripts were independently coded by both ERR and HBL to facilitate researcher triangulation. Discussions subsequently took place to assess consistency, compare and refine codes, and agree on the final coding framework. All remaining transcripts were then coded by ERR. Developed codes were categorised to form principle evolving themes. Recruitment, interviewing and analysis continued until theoretical saturation was reached, i.e. no significant new concepts emerged from interview analysis. Theoretical data saturation was reached after 15 interviews. Three additional interviews were conducted to confirm saturation had been achieved. The final themes and selection of appropriate supporting quotations were determined by consensus agreement between ERR and HBL.

## Results

### Participant characteristics

Eighteen VI adults participated in semi-structured interviews between October and December 2023 via either videoconference or telephone call. Duration of interviews ranged from approximately 18 to 156 min (median: 31.31 IQR: 18.67). Of the eighteen interviewees, ten were male and eight were female and classification of visual impairment ranged from B1 to B4. Acquired visual impairment applied to ten interviewees whereas eight interviewees have been VI since birth. Eleven interviewees could read braille. Further demographic information of the interviewees can be seen in Table 1.

**Table 1:** Demographic data of interviewees (n = 18)

Interviewee ID	Gender	Age (years)	Duration of VI	Vision classification^a^	Reads Braille	Education level^b^	Currently employed	Regular prescription medication
1	Man	55	15 years	B1	Yes	3rd Level	No	Yes
2	Woman	61	50 years	B1	Yes	3rd Level	Retired	Yes
3	Man	43	Since birth	B1	No	2nd Level	No	No
4	Woman	31	Since birth	B2	Yes	3rd Level	No	No
5	Man	49	Since birth	B1	Yes	3rd Level	Yes	Yes
6	Woman	55	Since birth	B2	Yes	3rd Level	Retired	No
7	Man	67	13 years	B3	No	3rd Level	No	Yes
8	Man	66	Since birth	B2	Yes	3rd Level	Yes	Yes
9	Woman	69	2 years	B3	Yes	3rd Level	Retired	Yes
10	Woman	74	12 years	B1	No	3rd Level	Retired	Yes
11	Woman	45	29 years	B1	Yes	3rd Level	No	Yes
12	Man	54	3 years	B3	No	2nd Level	No	Yes
13	Man	39	Since birth	B4	No	3rd Level	Yes	Yes
14	Man	74	Since birth	B1	No	3rd Level	Retired	Yes
15	Man	77	Since birth	B1	Yes	3rd Level	Retired	Yes
16	Man	30	28 years	B1	Yes	2nd Level	No	Yes
17	Woman	66	50 years	B1	Yes	3rd Level	Yes	Yes
18	Woman	33	Since birth	B4	No	3rd Level	No	Yes

^a^Categorised according to Vision Classification System from Vision Ireland. Further information can be found in Supplementary Materials

^b^Refers to Irish Educational System where Primary Level refers to Primary School education typically aged 4–12, Secondary Level refers to Secondary School education typically aged 13–18 and Third Level refers to University education

### Visually impaired adults experiences in community pharmacies

Four major themes emerged from the interviewee data: Staff Awareness, Information Accessibility, Physical Accessibility and Positive Supports. These were further divided into 9 subthemes as seen in Table 2. Explanation of the themes and supporting quotes are illustrated below.

**Table 2:** Themes and sub-themes resulting from thematic analysis of interview transcripts

Themes	Subthemes
Staff awareness	Recognition Poor communication
Medicines information accessibility	Medicines counselling and instruction Accessible formats Packaging identification
Physical accessibility of pharmacies	Internal layout External accessibility
Positive supports	Assistive technology Patient-staff relationships

#### Theme 1: Staff awareness

Interviewees generally reported insufficient disability awareness amongst pharmacy staff and necessity for disability awareness training or campaigns were highlighted. In some instances, staff were said to be unaware of correct protocols surrounding guide dogs. Pharmacy staff not identifying a VI patient had led to negative previous experiences and misunderstandings.

*‘I stood for about 5 min, with nobody coming near me, and you start wondering where I should start trying next’.*

*(Interviewee 15, Male)*

In certain cases, despite possessing a white cane, interviewees were not approached by staff when in need of assistance.

*‘To be aware with the white cane, that person may require additional service, to maybe just rethink it, to go up to them first rather than them having to come to look for somebody’*

*(Interviewee 13, Male)*

Lack of awareness from staff regarding how to best communicate with VI patients was a recurring problem for interviewees. An apparent discomfort amongst some staff regarding how to communicate or reluctance to do something which may be perceived as inappropriate was acknowledged. Interviewees found they generally had to explain their preferred communication method, while no interviewees had been queried regarding communication preferences.

*‘They literally handed me a pen and went tick the ones that you use regularly and I was like ‘ahh…’ So I actually I actually asked them ‘could you actually read them out’ and they were like ‘uh okay?’ They were quite hesitant about that. So, just kind of like a bit more awareness aware maybe, awareness is a huge thing’.*

*(Interviewee 18, Female)*

In some instances, staff elected to not speak directly to the VI patient. Opting instead to speak to a fully sighted person who accompanied them. Diminishing the VI adults’ independence.

*‘Handing my stuff to someone else and talking over me. Giving my change to someone else or leaving stuff on the counter. They usually hand your prescription to whoever’s with you and it would be tell her she needs to do this.’*

*(Interviewee 2, Female)*

Feelings of embarrassment, compromised confidentiality and privacy compared to fully sighted individuals due to poor communication methods were also highlighted. In very few instances’ had private conversations taken place in a consultation room.

#### Theme 2: Medicines information accessibility

Interviewees commonly reported problems being unable to access information about medicines. A lack of pharmacist knowledge regarding the significance of braille for identification purposes was emphasized. Most interviewees reported that braille instructions on medicines have been covered by a dispensing label. There were other instances where the text on labels was undersized and unclear. Some interviewees had requested notes on their patient files to stop this occurring.

*‘All the time, a lot of medicines generally have the printed label over the Braille. If you don’t go to the chemist yourself, you will run into problems. Because while I’ve insisted there is a note on my file saying do not cover the braille label. If somebody else picks up my prescription. It’ll be covered’.*

*(Interviewee 2, Female)*

The importance of consistent medicines packaging such as keeping products in original containers for identification purposes was also highlighted.

*‘You have the packet and then they’re put into the Ziplock bags. You can’t distinguish which is which with the packets, whereas if you’re in the box, you can say yeah, these are paracetamol. You don’t have to get anyone to tell you what they are. I find those little see through bags annoying’.*

*(Interviewee 3, Male)*

When receiving new prescription medicines, particularly inhalers or eyes drops, in some instances interviewees received very little information about how to use the product. Instead, being referred to the product leaflet or told to “take as directed”.

*‘When I was first prescribed my Ventolin inhaler, I found they were saying that’s all on the leaflet. I was like, but you need to show me how to use it. I ended up having to get somebody who’s on the same inhaler to show me how to use it. I did mention it to my GP and haven’t used that pharmacy since’.*

*(Interviewee 4, Female)*

In general, obtaining medicines information in alternative or accessible formats proved challenging. While in some instances pharmacists had successfully obtained accessible forms, in general pharmacist knowledge regarding how to obtain alternative formats was lacking. Interviewees expressed interest in being provided alternative resources and had previously sourced medicines information themselves online.

*‘He knew nothing about the availability of Braille leaflets. The leaflets that he was going to give me in [the pharmacy] were available in Braille. He did everything he could. He got them in the end’.*

*(Interviewee 17, Female)*

#### Theme 3: Physical accessibility

Interviewees spoke of challenges navigating the internal environment of pharmacies. Depending on the layout, particularly in larger premises, locating the correct counter was difficult.

*‘They had moved the shop around and I didn’t know. I actually ended up behind the counter because I didn’t actually know, they had moved the counter. I was treated as if I had done it deliberately.’*

*(Interviewee 17, Female)*

Some layouts were not judged to follow a logical path and unexpected updates instore led to confusion. In smaller premises, navigating narrow aisles posed a major challenge, particularly with guide dogs. Poor lighting and various physical obstacles, caused access problems.

‘*Steps and narrow aisleways, or stuff in the aisleways, like boxes on the ground or the bargain basin at the end, those kinds of things give you bad access.’*

*(Interviewee 1, Male)*

Regarding external accessibility of pharmacies, the presence of the stereotypical green crosses was highlighted as a positive means of identification. In contrast, presence of steps were an accessibility obstacle and concerns regarding large paned glass shop fronts were raised, as these can resemble entrances. Interviewees highlighted automatic doors, handrails, ramps and proximity of disabled parking and public transport as accessibility enablers. Staff had also previously accompanied patients to nearby public transport. Overall, challenges with external accessibility were less prominent than internal.

#### Theme 4: Positive supports

Despite challenges, supportive measures implemented by pharmacy staff were evident. While these measures were commended, scope for improvement was articulated. Examples of current aids for medication management included blister packs, measured syringes, cutting tablets in half and placing medications in different sized or separate containers. Regarding utilisation of assistive technology, mobile applications were discussed by some interviewees. More widespread use of applications such as NaviLens®, SeeingAI® and Be My Eyes® was suggested. A clear emphasis was placed on increased collaboration with staff to obtain full benefit.

*‘NaviLens® works very well for navigation. The amount of information that can be put into this is huge, because it is a QR code. A pharmacist can have that ‘the register is 2 metres to right and we have such and such on offer this week’*

*(Interviewee 1, Male)*

*‘We open our iPhone and press Be My Eyes®. It has a new feature, which is called ‘take picture’. It will give you all the information on the package and if that’s not good enough, you can look for more help from a volunteer’.*

*(Interviewee 2, Female)*

During interviews the perceived correlation between close patient-staff relationships and improved service was evident. A preference to attend a single pharmacy was highlighted with the primary motivation being to establish this connection. Lower standards of service were generally experienced in unfamiliar pharmacies. Interviewees encouraged staff to learn their patients’ names and to use them frequently.

*‘I make a point for my own access of talking to people on tills and say ‘what’s your name?’ and just make the connection and then you’re fine. The treatment is a bit better.’*

*(Interviewee 2, Female)*

According to interviewees using a person’s name positively contributes to increased comfort and facilitates directional guidance around the pharmacy.

*‘If you do know the customer’s name, use it. That just re-assures the person, there is nothing more embarrassing if they say ‘what can I do for you?’ and you answer, and they’re talking to the person next to you, there is more to it but it’s just a bit embarrassing as well.’*

*(Interviewee 8, Male)*

## Discussion

### Statement of key findings

This study highlights that at present communication between community pharmacy staff and VI patients is generally unstructured and lacking. Improved awareness and staff training surrounding both the identification of VI patients and their specific needs is required. Previous international studies investigating pharmacist views have highlighted similar difficulties in identifying VI patients and a lack of training available. Significantly, during the interviews it was revealed that VI patients highly value personal relationships with pharmacy staff. These relationships, when developed, can aid in attainment of an improved sense of independence and privacy for this cohort. Pharmacy staff should both be encouraged and provided with necessary guidance and training, to communicate effectively with VI patients.

It is clear from this study that communication difficulties between pharmacy staff and VI patients are not just limited to verbal interactions within the community pharmacy setting. Provision of print-form medicines information and packaging were also highlighted by interviewees as often being inaccessible and inappropriate for this cohort. Therefore, to ensure the safe communication of medicines information pharmacists and pharmacy technicians should ensure; braille text is never covered with dispensing labels, consistent packaging and capacity to access alternative formats, if requested.

### Strengths and weaknesses

This present study provides the first qualitative analysis of VI patients views regarding community pharmacy care in Ireland. This research is limited to the views of interviewees from Ireland. However, through the diversity of interviewee demographics and by setting the focus of the interviews on experiences visiting community pharmacies, international stakeholders can consider these results and apply them in their own contexts. Adults of all ages (18 years and older) were permitted for inclusion to ensure a wide range of experiences across the lifespan were obtained. The interviewer was a fifth-year pharmacy student (ERR) with experience working in a community pharmacy, which may have aided in interpreting some interviewee comments. To minimise any potential biases self-reflection was conducted after each interview to ensure objectivity in data collection. A notable strength of the study was the consistency in data collection and analysis, as all interviews were conducted by the same researcher (ERR), facilitating a uniform approach to questioning. This qualitative study was reported following the SPRQ checklist and there was strict adherence to the six phases of Braun and Clarke thematic analysis. Vision Ireland ensured accessibility of all study materials and aided with study recruitment. However, it is acknowledged that recruitment through this organisation may have identified interviewees with strong views on this topic who were more likely to participate.

### Interpretation

This work has identified distinct areas in which community pharmacy practice should be adapted to improve experiences of VI patients. It was seen that through mostly minor and reasonable adjustments to usual practice, improvements are possible. Need for such changes were identified across the four themes which emerged from these interviews i.e. staff awareness, medicines information accessibility, physical accessibility of pharmacies and positive supports.

From a patient perspective it appears that pharmacy staff typically appear unsure or unprepared regarding how to best communicate with these patients. While a perceived lack of available training in this area internationally has been highlighted previously [12, 14–17], it is clear a similar problem exits in Ireland. Locally speaking there is a distinct lack of contemporary guidance documents available to Irish pharmacy staff on how to care for and identify VI patients. While the Equality Authority and Irish Pharmacy Union have previously released guidance documents, these are outdated and are not specific to VI patients [23, 24]. Instead, these provide general advice regarding patients with disabilities. However, even within the VI cohort, needs and preferences for communication and service differ significantly, and are different to those with other sensory impairments. Thus, a more tailored and direct approach to guidance and recommendations is required. It seems that internationally the availability of guidance for pharmacy teams differs significantly across jurisdictions and difficulties surrounding VI patients not disclosing their impairment, hindering identification has been discussed previously [14, 15, 17, 25]. Early educational interventions for pharmacy students, such as in Australia, appear to significantly improve knowledge in this area, but appear limited in implementation [26].

The development of close professional relationships between pharmacy staff and VI patients was identified by interviewees as a method to overcome the perceived lack of independence or privacy experienced by this cohort. This can be as simple as directly addressing the patient rather than a fully sighted person accompanying them or calling them by name to avoid confusion. Previous research has identified differing views from pharmacists on reliance on caregivers for VI patients, while it was also noted that communicating with proxies may lead to ultimate information provided to VI patients being incomplete or distorted [2, 12, 15]. In the current study, another method to increase independence of VI patients was suggested by patients of different ages. This involves adoption of assistive technologies, including those tailored to relay medicines information, or to aid navigation in the pharmacy, as identified in previous literature [27, 28]. Aside from such applications, this study also identified the need for generally improved accessibility of internal pharmacy environments for VI patients. This was also previously highlighted by both patients with sensory impairments and pharmacy personnel in Scotland [14, 16]. In Ireland, the pharmacy regulator has released guidelines on pharmacy premises requirements including reference to disability access [29]. However, despite this, as observed obstacles for accessibility remain for VI patients.

Provision of medicines information was also identified as an area where improved accessibility for VI patients is required. In line with previous findings [2, 16, 30–32], interviewees provided examples of poor practices, including braille on packaging covered with dispensing labels, inappropriate label font size, medications being removed from original packaging or pharmacists being unaware of alternative information formats. Despite existence of labelling guidelines internationally it appears that deficiencies in the provision of medicines information such as those identified here are commonly seen. Accordingly, VI patients are currently being put at higher risk of inappropriate medicines use and suboptimal therapeutical outcomes owing to confusion with medicines identification and counselling. Work is required to highlight these issues to pharmacy staff.

### Further research

This research marks an important advancement in understanding the current level of care provided at community pharmacies to VI patients. However, to develop tailored guidance and improve awareness of pharmacy staff to necessary improvements, the perspectives of the community pharmacy staff in Ireland should first be elucidated, to learn their perspectives and learning priorities. Only then can work commence on updated tailored recommendations and guidance campaigns for pharmacy teams which consider both patient views and environmental constraints.

## Conclusion

This study reveals invaluable insights into the current care provided to VI patients at community pharmacies. While in some instances substantial changes to practice are required, across the interviews, minor recommendations based on personal experiences were made. These will help to increase pharmacy accessibility and provide improved care for this cohort when visiting. Honest and open communication and relationship building will aid in identifying how best to serve their needs. An openness to listen and learn will prove essential moving forward to ensure community pharmacies are inclusive environments.

## Supplementary Information

Below is the link to the electronic supplementary material.

Supplementary file1 (PDF 103 KB)

Supplementary file2 (PDF 167 KB)
