# Supplementary material for: Opinions of visually impaired adults on the care provided at community pharmacies: a qualitative interview study
Source: Int J Clin Pharm. 2025 Mar 13;47(3):854–62. doi: 10.1007/s11096-025-01888-1 (PMC12125151; doi:10.1007/s11096-025-01888-1)
Supplement: Supplementary file 1 — Supplementary file1 (PDF 103 KB) [file 11096_2025_1888_MOESM1_ESM.pdf]

Supplementary Materials 1

Title: Opinions of Visually Impaired Adults on the Care Provided at Community Pharmacies: A Qualitative Interview Study.

Journal: International Journal of Clinical Pharmacy

Ellen Roche Ryan, Harriet Bennett-Lenane*.

School of Pharmacy, University College Cork, Cork, Ireland.

*Corresponding Author, email: hbennettlenane@ucc.ie

Vision Classification System

The below Vision Classification System used by Vision Ireland is based on the International Blind Sports Federation (IBSA) Classification for Athletes with Visual Impairment. Categories B1 – B3 may be referred to as “legally blind” or “registered blind”.

Classification	Term	Description
**B1**	No Vision	No perception of light, ability to detect hand movements, but unable to recognise shapes.<br>Logarithm of the Minimum Angle of Resolution (LogMAR): < 2.6
**B2**	Low Vision	Able to count fingers (at 15 cm) to a visual acuity of up to and including 2/60, OR a visual field of under 10 degrees (even if their acuity is better than 2/60)<br>LogMAR 1.50 to 2.6
**B3**	Low Vision	Visual Acuity of 2/60 to 6/60, and/or a visual field of less than 40 degrees.<br>LogMAR I to 1.40
**B4**	Useful Vision	Anyone with better vision than 6/60, and up to and including 6/24.<br>LogMAR 0.6 to 1.0
**B5**	Useful Vision	Visual acuity of better than 6/24, but not better than 6/18.<br>LogMAR 0.6 to 0.5
**Unsure/Unable to Determine**		
**Prefer Not to Say**		
