# Supplementary material for: Opinions of visually impaired adults on the care provided at community pharmacies: a qualitative interview study
Source: Int J Clin Pharm. 2025 Mar 13;47(3):854–62. doi: 10.1007/s11096-025-01888-1 (PMC12125151; doi:10.1007/s11096-025-01888-1)
Supplement: Supplementary file 2 — Supplementary file2 (PDF 167 KB) [file 11096_2025_1888_MOESM2_ESM.pdf]

Supplementary Materials 2

Title: Opinions of Visually Impaired Adults on the Care Provided at Community Pharmacies: A Qualitative Interview Study.

Journal: International Journal of Clinical Pharmacy

Ellen Roche Ryan, Harriet Bennett-Lenane*.

School of Pharmacy, University College Cork, Cork, Ireland.

*Corresponding Author, email: hbennettlenane@ucc.ie

Semi-Structured Interview Topic Guide including Main and Follow-Up Questions.

Background/Overall Experience

1. Can you describe what you use community pharmacies for and how often you visit?
2. Could you describe your general experience of visiting pharmacies in Ireland?
3. Can you share an example of any previous positive or negative experience when visiting a pharmacy?

Barriers or Challenges Experienced

4. What, if anything, makes it difficult or challenging for you when visiting a community pharmacy?
5. What difficulties, if any, have you experienced with the external or internal physical accessibility/layout of pharmacies?
6. Have you ever had difficulty in accessing information or advice related to a product, medication, or pharmacy service? If so, can you elaborate on the reasons for this?

Staff

7. Have you experienced any situations of particularly good or poor communication from pharmacy staff? If so, could you please provide an example?
8. Do you believe there is enough awareness amongst pharmacy staff regarding needs of patients with visual impairment? Why or why not?
9. Do you typically feel comfortable asking pharmacy staff for advice? Is there a particular reason for this?

Accessible Materials/Aids

10. Is there anything your pharmacist or pharmacy staff do that makes it easier or harder for you when you visit?
11. If you receive any over the counter or prescription medications from a pharmacy, are there any additional solutions/aids provided to you to improve your medication use? If so what?

12. Can you describe any problems with medication labelling/packaging which may affect your ability to use them correctly?
13. Do pharmacies typically provide informational materials in accessible formats? Do you have any example of this?

Suggestions

14. Tell me about what key areas community pharmacies in Ireland should focus on to improve their service of patients with visual impairments?
15. What advice would you give to pharmacy staff to better accommodate patients with visual impairments?

Closing

16. Is there anything else that we have not yet talked about that you think would be useful or good to discuss?
